# A Synthetic Sponge System Against miRNAs of the miR-17/92 Cluster Targets Transcriptional MYC Dosage Compensation in Aneuploid Cancer

**DOI:** 10.3390/cells14171384

**Published:** 2025-09-04

**Authors:** Diana M. Bravo-Estupiñan, Carsten Geiß, Jorge L. Arias-Arias, Mariela Montaño-Samaniego, Ricardo Chinchilla-Monge, Christian Marín-Müller, Steve Quirós-Barrantes, Anne Régnier-Vigouroux, Miguel Ibáñez-Hernández, Rodrigo A Mora-Rodríguez

**Affiliations:** 1 Programa de Doctorado en Ciencias, Sistema de Estudios de Posgrado (SEP), Universidad de Costa Rica, San José 20601, Costa Rica; diana.marcela.bravo94@gmail.com; 2 Laboratorio de Quimiosensibilidad Tumoral (LQT), Centro de Investigación en Enfermedades Tropicales (CIET), Facultad de Microbiología, Universidad de Costa Rica, San José 20601, Costa Rica; jorgeluis.arias@ucr.ac.cr (J.L.A.-A.); steve.quiros@ucr.ac.cr (S.Q.-B.); 3 Laboratorio de Terapia Génica, Departamento de Bioquímica, Escuela Nacional de Ciencias Biológicas, Instituto Politécnico Nacional, Carpio and Plan de Ayala, Col. Sto Tomás, Miguel Hidalgo, Ciudad de México 11340, Mexico; mariela.mont3091@gmail.com (M.M.-S.); mibanez@ipn.mx (M.I.-H.); 4 CICICA, Centro de Investigación en Cirugía y Cáncer, Universidad de Costa Rica, San José 20601, Costa Rica; ricardo.chinchilla_m@ucr.ac.cr; 5 Centro Nacional de Innovaciones Biotecnológicas (CENIBiot), CeNAT-CONARE, Pavas, San José 10109, Costa Rica; 6 Institute for Developmental Biology and Neurobiology, Johannes Gutenberg University, 55128 Mainz, Germany; vigouroux@uni-mainz.de; 7 Laboratorio de Técnicas Fototérmicas, Departamento de Ciencias Básicas, Unidad Politécnica Interdisciplinaria de Biotecnología, Instituto Politécnico Nacional, Ciudad de México 07340, Mexico; 8 Speratum Biopharma, Inc., Centro Nacional de Innovación Biotecnológica Nacional (CENIBiot), San José 20601, Costa Rica; christian@speratum.com

**Keywords:** microRNA sponge, MYC, gene dosage compensation, genomic instability, miR-17/92 cluster, cancer gene therapy, miRNA inhibition, aneuploidy, synthetic biology, tumor suppressor regulation

## Abstract

Background: Genomic instability, a hallmark of cancer, leads to copy number variations disrupting gene dosage balance and contributing to tumor progression. One of the most affected oncogenes is MYC, whose overexpression is tightly regulated to avoid cytotoxicity. In aneuploid cancer cells, gene dosage compensation mechanisms involving microRNAs (miRNAs) from the miR-17/92 cluster contribute in regulating MYC expression. Targeting this miRNA-mediated compensation system represents a promising therapeutic strategy leading to an uncontrolled and lethal MYC overexpression. Results: Synthetic miRNA sponges targeting miR-17, miR-19a, and miR-20a, key regulators of MYC dosage compensation, were designed and validated. Breast cancer cells (MCF7) with stable exogenous MYC overexpression were used to assess the impact of sponge constructs on MYC regulation. Quantitative RT-PCR revealed a significant reduction in miRNA expression and a corresponding increase in endogenous MYC levels upon sponge treatment. Functional assays in multiple colorectal cancer cell lines with varying MYC copy numbers demonstrated a time-dependent increase in cell death following sponge transfection. Cytotoxic effects increased with MYC copy number, confirming a correlation between gene dosage sensitivity and therapeutic response. Conclusions: Our findings demonstrate that miRNA sponges targeting the miR-17/92 cluster can effectively disrupt MYC dosage compensation, leading to selective cytotoxicity in MYC-amplified cancer cells.

## 1. Introduction

Cancer is a complex group of diseases characterized by uncontrolled cell growth, potential metastasis, and increased risk of death. These diseases arise from a combination of genetic, epigenetic, and metabolic alterations that enable cancer cells to evade normal regulatory mechanisms [1,2]. Genomic instability, a hallmark of cancer, drives the accumulation of mutations, chromosomal rearrangements, and other DNA alterations, fueling tumor heterogeneity, and enabling the emergence of the remaining cancer hallmarks contributing thereby to therapeutic resistance [3,4,5]. Indeed, despite advances in conventional therapies, such as chemotherapy, radiotherapy, and targeted agents, treatment outcomes remain limited by drug resistance [6,7], toxicity, and tumor heterogeneity. A critical challenge lies in the regulation of oncogenes such as MYC, a master transcription factor that controls cell growth and proliferation. MYC is frequently amplified in cancer [8], and its overexpression can drive tumor progression, although excessive MYC levels may also trigger apoptosis [9]. Cells with MYC amplification rely on compensatory mechanisms, including regulation by specific microRNAs, to maintain oncogene expression within a tolerable range.

The miR-17/92 cluster, also called Onco-miR-1, is a polycistronic microRNA cluster located on chromosome 13q31.3 that encodes six mature miRNAs (miR-17, miR-18a, miR-19a, miR-20a, miR-19b-1, miR-92a-1), many of which have been implicated in oncogenic processes [10]. In addition to these functions, the miR-17/92 cluster has been shown to regulate the maintenance, self-renewal, and tumorigenic potential of cancer stem-like cells, suggesting a critical role in the stem cell origin of tumors [11,12,13]. By controlling both proliferation and stem cell-like properties, this cluster contributes to tumor initiation, progression, and heterogeneity, highlighting its relevance as a therapeutic target. This miR-17/92 cluster has also been identified as a key regulator of MYC expression dosage, influencing cell proliferation and survival. Our previous study [8] provided strong experimental and computational evidence that the miR-17/92 cluster plays a central role in MYC dosage compensation in aneuploid cancer cells. By integrating data from 1000 cell lines of the Cancer Cell Line Encyclopedia (CCLE) and 979 breast cancer samples from The Cancer Genome Atlas (TCGA), we validated this mechanism and established a solid conceptual framework. Targeting this regulatory system with miRNA-based approaches, such as sponge constructs, represents a promising but underexplored strategy to simultaneously destabilize MYC dosage compensation and impair cancer cell survival.

Our previous findings not only confirmed the biological relevance of MYC dosage compensation but also raised the question if miRNA sponges targeting the miR-17/92 cluster can overcome MYC dosage compensation and induce selective cytotoxicity in MYC-amplified cancer cells. We hypothesize that inhibition of key miRNAs within the miR-17/92 cluster (miR-17, miR-19a, and miR-20a) using sponge constructs will destabilize the MYC dosage compensation system, unleash cytotoxicity and reduce tumor cell viability depending on MYC expression levels.

The miR-17/92 cluster has been the focus of extensive research as a therapeutic target in cancer treatment [9]. A promising strategy involves the use of LNA gapmeRs (Locked Nucleic Acid gapmers), which are antisense oligonucleotides designed to induce the specific degradation of messenger RNA (mRNA) or non-coding RNA through RNase H activation; for example, gapmeR MIR17PTi targets and degrades pri-miR-17-92, thereby blocking the biogenesis of all microRNAs within the cluster. This approach has demonstrated preclinical efficacy in multiple myeloma models, inducing apoptosis in tumor cells through the deregulation of MYC-regulated circuits and the activation of pro-apoptotic genes such as BIM [14]. In mantle cell lymphoma, inhibition of the miR-17/92 cluster has been shown to reduce mTORC1 signaling by restoring the activity of key upstream regulators such as LKB1, TSC1, and AMPK, suggesting that overexpression of this cluster contributes to the activation of critical oncogenic pathways [15]. Furthermore, in medulloblastoma models, the use of LNA anti-miRs targeting miR-17 and miR-19 has resulted in a significant reduction in tumor growth both in vitro and in vivo, with no evidence of systemic toxicity [16].

However, the polycistronic nature of the miR-17/92 cluster, which encodes six mature miRNAs with overlapping yet distinct oncogenic functions, complicates therapeutic targeting using conventional antisense oligonucleotides that typically silence only individual miRNAs. Moreover, the redundancy of MYC dosage compensation—mediated by at least three different miRNAs from the miR-17/92 cluster [8] requires the development of specialized strategies capable of simultaneously inhibiting multiple miRNAs while ensuring selective cytotoxicity in MYC-amplified cancer cells. Given this context, we designed and validated miRNA sponge systems capable of disrupting MYC dosage compensation in aneuploid breast cancer cells.

In parallel, although MYC dosage compensation has emerged as a promising therapeutic target for various aneuploid cancers with increased MYC copy numbers, its effective targeting is limited by the lack of robust cellular models to test candidate molecules capable of disrupting this compensatory mechanism. Therefore, we propose the use of a genetic tug-of-war established in a stable cellular system to enable the study of gene dosage compensation at the RNA level. Moreover, the use of cancer cell lines with varying MYC copy numbers reveals the dosage-dependence of the cytotoxicity induced by the interruption of the miR-17/92-mediated regulatory loops of MYC expression. Understanding the interplay between genomic instability, MYC dosage compensation, and the underlying interactions with the miRNA-17/92 not only sheds light on cancer pathogenesis but also highlights potential therapeutic targets to disrupt these compensatory mechanisms and impair tumor growth.

## 2. Materials and Methods

### 2.1. Experimental Model

The human breast cancer cell line MCF7 and the colon cancer cell lines SW-620, HCT-15, and HT-29 were obtained as part of the NCI60 collection (Developmental Therapeutics Program, NIH, Bethesda, MD, USA). All cell lines were cultured in RPMI 1640 medium (Roswell Park Memorial Institute, Buffalo, New York, NY, USA) supplemented with 10% fetal bovine serum (FBS, Gibco^®^, Thermo Fisher Scientific, New York, NY, USA) antibiotic-antimycotic B (penicillin, streptomycin, and amphotericin; Gibco^®^ Thermo Fisher Scientific, New York, NY, USA), and GlutaMAX™ (Gibco^®^ Thermo Fisher Scientific, New York, NY, USA), an optimized alternative to L-glutamine. Cells were maintained at 37 °C in a humidified atmosphere with 5% CO_2_.

### 2.2. Method Details

#### 2.2.1. Design and Composition of the Anti-miR-17, Anti-miR-19a and Anti-miR-20a Sponge

The sequence of the anti-miR-17, anti-miR-19a and anti-miR-20a sponge was designed using the miRNAsong software version 2.0.0 [17], consisting of antisense oligonucleotides specific to anti-miR-17, anti-miR-19a and anti-miR-20a, located in tandem and separated by a specific spacer region between each anti-miRNA sequence. The 5′ and 3′ ends of the anti-miRNA sponge sequence correspond to specific recognition sites for the restriction enzymes XbaI and NcoI at each end respectively, for subsequent cloning into the vector.

#### 2.2.2. In Silico Analysis of the Specificity and Functionality of the Sponge

To assess the specificity and binding efficiency of the anti–miR-17, anti–miR-19a, and anti–miR-20a sponge sequences, the miRNAsong software version 2.0.0 was used [17]. For this in silico analysis, each sponge sequence corresponding to each anti-miR was individually loaded into the program, validated, and evaluated.

#### 2.2.3. Genetic Vector Design and in Silico Construction

For the design of the genetic vector, SnapGene version 8 was used, starting with the eukaryotic expression vector pSBbi––mCardinal–P2A-T2A-Puro, with a size of 6.7 Kb. The in silico construction was carried out by releasing the MCS fragment with the restriction enzymes XbaI and NcoI and ligating the fragment of interest (anti miR-17, anti miR-19a and anti miR-20a sponge) to provide directionality for cloning in the vector.

#### 2.2.4. Molecular Synthesis and Characterization of the Recombinant Plasmid PSBbi–AntimiR-17–AntimiR-19a–AntimiR-20a–mCardinal–P2A-T2A-Puro

The recombinant plasmid (pSBbi–antimiR-17–antimiR-19a–antimiR-20a–mCardinal– P2A-T2A-Puro) designed in silico was commercially synthesized and cloned by GenScript (GenScript, Piscataway, NJ, USA).

The concentration and purity of pSBbi–antimiR-17–antimiR-19a–antimiR-20a–mCardinal–P2A-T2A-Puro was validated using a Nanodrop 1000TM spectrophotometer (Thermo ScientificTM). The integrity of the plasmid insert was confirmed using Sanger sequencing. The obtained sequence was used to perform a local alignment analysis with the EMBOSS Water online software (https://www.ebi.ac.uk/Tools/psa/emboss_water/, accessed on 18 February 2025), comparing it with the sequence of the recombinant plasmid.

#### 2.2.5. Generation of Stable Cell Lines

To validate the implementation of the tug-of-war technique, a cell line expressing an additional codon optimized endo MYC sequence was generated as described previously [8]. Therefore, 1 × 10^6^ MCF7 cells per well were seeded in a 6-well plate in 2 mL RPMI medium supplemented with 10% of FBS (Gibco^®^), and incubated for 24 h at 5% CO_2_ and 37 °C. The cell line was co-transfected with pSBbi-moxGFP-P2A-T2A-cMYC-Puro (Addgene plasmid # 176892) and pCMV (CAT)T7-SB100 (Addgene plasmid # 34879) using Lipofectamine^®^ 3000 transfection reagent (Thermo Fisher Scientific) optimized protocol for stable cell lines preparation. 24 h post-transfection, stable expression was evaluated by fluorescence microscopy and stable cells were selected by cultivating the cells for 15 days in 2 µg/mL puromycin containing medium.

#### 2.2.6. Flow Cytometry

MCF7 cells with stable exoMYC expression were trypsinized, collected, and washed twice with phosphate-buffered saline (PBS). Cells were resuspended in PBS and stained with propidium iodide (PI, Invitrogen, Thermo Fisher Scientific, Carlsbad, CA, USA) at a 1 mg/mL. The suspension was incubated for 30 min at 37 °C in the dark. Flow cytometry was performed using a CytoFLEX flow cytometer (Beckman Coulter Life Sciences, Brea, CA, USA) equipped with a 488 nm excitation laser. Cell populations were identified based on forward scatter area (FSC-A) versus side scatter area (SSC-A) parameters to exclude debris. GFP expression was measured in the FITC-A channel, and PI staining was detected in the Y585-PE-A channel to distinguish viable from non-viable cells. Data was acquired and analyzed using CyExpert software version 2.4.0.28 with standardized gating strategies.

#### 2.2.7. Sponge Transfections and Cytotoxicity Assays

To evaluate the effect of the sponge sequence, MCF7, HCT15, HT29 and SW620 cell lines were seeded (15,000 cells per well in 6-well plates) in RPMI medium supplemented with 10% of FBS (Gibco^®^), and incubated for 24 h at 5% CO_2_ and 37 °C. Transfection was carried out according to the protocol established for Lipofectamine 3000 (Thermo Fisher Scientific). In this study, we included a positive control (sponge vector: pSBbi–antimiR-17–antimiR-19a–antimiR-20a–mCardinal–P2A-T2A-Puro) (see Figure S1 for plasmid maps) and a negative control (empty vector: pSBbi–mCardinal–P2A-T2A-Puro) to assess the specific effects of the miRNA sponge on gene expression and cellular responses. The lipoplex solution formed in OptiMEM was added to each well, incubating for 4 h under the same culture conditions. Cytotoxicity was assessed using Sytox Green (Invitrogen S11381). Following transfection, cells were incubated with Sytox Green (10 mg/mL) to label non-viable cells, which were subsequently visualized by fluorescence imaging. The number of Sytox Green–positive cells was quantified and compared to the total number of cells per well, allowing the calculation of the cytotoxicity percentage as the ratio of dead cells to the total cell population.

#### 2.2.8. Automated Fluorescence Microscopy Assay

Fluorescence imaging was performed using a Cytation 5 automated fluorescence microscope (BioTek, Vermont, United States). After transfection, cell nuclei and dead cells were stained by incubating the cultures for 30 min with Hoechst 33342 (1.25 mg/mL, Invitrogen H3570) and Sytox Green (10 mg/mL, Invitrogen S11381) in complete RPMI medium. The cells were subsequently maintained inside the Cytation 5 imaging chamber under controlled conditions (37 °C, 5% CO_2_) for 72 h. Images were acquired every 12 h in the blue (Hoechst), green (Sytox Green), and red (mCardinal) fluorescence channels. Quantification of live and dead cells was carried out using CellProfiler image analysis software number (SCR_007358), with a customized pipeline designed to count total nuclei and Sytox Green–positive cells. Cytotoxicity percentage was determined by dividing the number of Sytox Green–positive cells by the total number of nuclei in each well.

#### 2.2.9. RNA Extraction and RT-qPCR

To isolate total RNA from cells, the PureLink™ RNA Mini Kit (Thermo Fisher Scientific #12183018A) was used. The concentration and purity of the RNA was determined using the RNA Quantification, broad range assay kit (Thermo Fisher Scientific #Q10210) and the structural integrity was determined using the Qubit™ RNA IQ Assay Kits (Thermo Fisher Scientific #Q33221) according to manufacturer’s recommendations and setting up recommended standards for RNA quantification and structural integrity.

Reverse transcription and qPCR were performed in one step using the following primers: GAPDH forward: 5’-CTCAGACACCATGGGGAAGG-3’, reverse: 5’-TTCCCGTTCTCAGCCTTGAC-3’ and probe: VIC- GAGTCAACGGATTTGGTCGT; MYC Endo forward: 5’-AAACTTTGCCCATAGCAGCG-3’, reverse: 5’-CGGGAGGCTGCTGGTTTT-3’ and probe: VIC-CTGAAAGGCTCTCCTTGCAG; MYC Exo forward: 5’-GGCCGTGACACCTTTCAGCC-3’, reverse: 5’-TCGGACACCAGCTTTGCTGC-3’ and probe: 6FAM-ACAGAGCTGCTGGGCGGCGA.

For the miRNA expression analysis, the SuperScript^®^ III Platinum^®^ SYBR^®^ Green One-Step qRT-PCR Kit (ThermoFisher Scientific, Waltham, MA, USA) was used, performing three technical replicates and three biological replicates. The primers for miRNA analysis were as follows: SNORD110 forward 5′-ACGCAATCACTGATGTCTC’, reverse 5′-AGTTTTTTTTTTTTTTTGCTCAGA-3′, hsa-miR-17-5p forward 5′-GCAAAGTGCTTACAGTGC-3′, reverse 5′-TCCAGTTTTTTTTTTTTTTTCTACC-3′, hsa-miR-19a-3p forward 5′-GCAGTGTGCAAATCTATGC-3′, reverse 5′-GGTCCAGTTTTTTTTTTTTTTTCAG-3′ and hsa-miR-20a-5p forward 5′-GCAGTAAAGTGCTTATAGTGC-3′, reverse 5′-GTCCAGTTTTTTTTTTTTTTTCTACC-3′. For miRNA data normalization, SNORD110 was utilized. The thermal cycling protocol was followed as the manufacturer indicates and by 40 cycles, Ct’s values were collected and analyzed.

Gene or miRNA expression levels were quantified through relative quantification (RQ) employing the 2^−ΔΔCt^ method, as described by Livak and Schmittgen in 2001 [18]. In the case of mRNA analysis, GAPDH was used as reference gene.

#### 2.2.10. Statistical Analysis

Statistical analysis was performed using GraphPad Prism 8.0.1 software. T-tests were performed to analyze means of samples with a significance defined by an α of 0.05. The resulting *p*-values were corrected for multiple comparisons using the Holm-Sidak method. Figure legends contain the details for each of the corresponding statistical analysis.

## 3. Results

### 3.1. A Stable Genetic Tug-of-War System Enables the Study of MYC Dosage Compensation in Breast Cancer Cells

In our previous report we developed a framework to identify gene dosage compensation at the transcriptional level using an exogenous version of MYC (exo MYC) that incorporates a sequence with optimized codons, allowing it to be distinguished from endogenous human MYC [8]. Additionally, the exo MYC sequence was modified to eliminate the UTR sequence to prevent transcriptomic regulation by miRNAs. However, in that system, the exo MYC expression was transient, precluding a detailed characterization of the gene dosage compensation mechanism and limiting the assessment of potential therapeutic strategies to target MYC dosage compensation.

**Figure 1:**
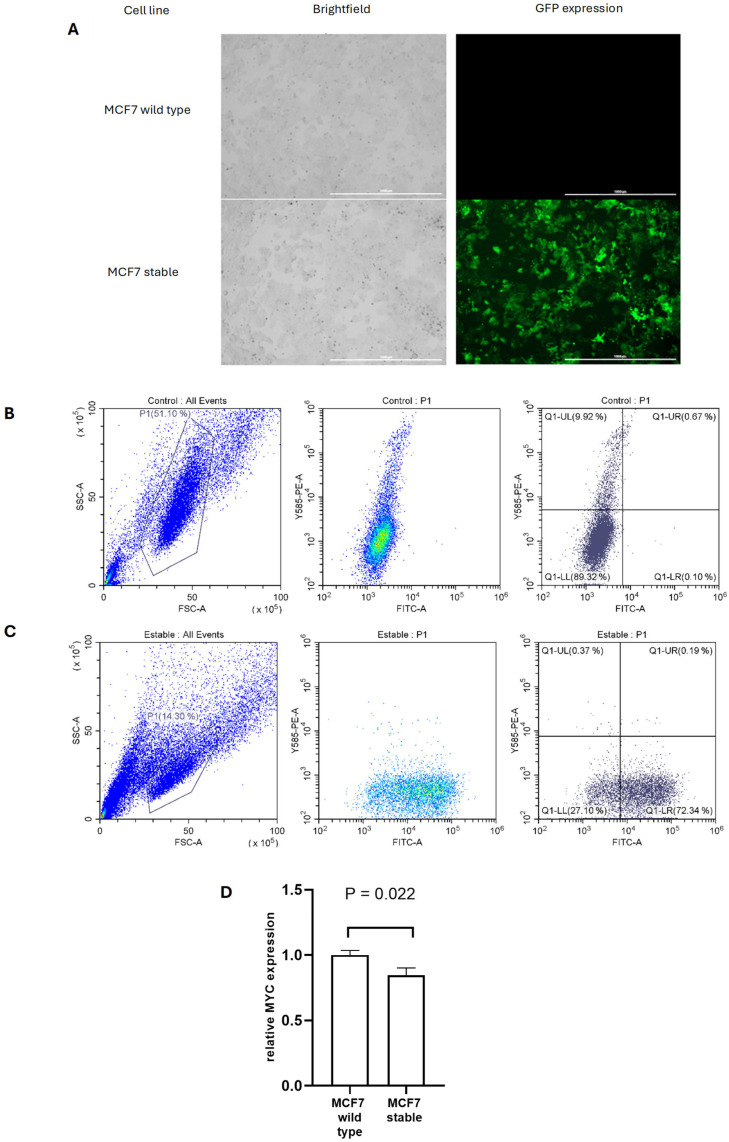
Expression of the GFP Reporter Gene in MCF7 Cells with Stable Exogenous MYC Expression. (**A**) Expression of the GFP reporter gene in MCF7 wild type and MCF7-exoMYC lines 15 days post-transfection. GFP protein expression is observed, indicating stable expression of the exogenous MYC gene. Scale bar: 1000 µm. (**B**,**C**) Dot Plot of the MCF7 Control (wild type) Cell Line and MCF7-exoMYC line showing the percentages of GFP positive cell population. The selected events for analysis are displayed, with the cell population delineated by the P1 gate. Additionally, the respective quadrants show the distribution of control cells based on GFP expression and PI staining. Quadrants from upper left to lower left: Q1: PI+/GFP−; Q2: PI+/GFP+; Q3: PI-/GFP+; Q4: PI−/GFP−. (**D**) Relative mRNA Expression Levels of Endogenous MYC for Each Cell Line. A paired *t*-test was conducted, which revealed significant differences in the relative expression of endogenous MYC between MCF7 wild-type and MCF7-exoMYC lines, with a *p*-value of 0.022.

To investigate gene dosage compensation in a stable setting, we employed the MCF7 breast cancer cell line and introduced exogenous MYC using a plasmid system previously described [8,19]. The strategy involved co-transfecting cells with the MYC-expressing construct and a transposase-encoding plasmid to facilitate genomic integration. Successful incorporation and expression of the construct were indicated by GFP fluorescence. Transfected cells were monitored over time using the BioTek Cytation 5 multimode reader, allowing the identification of stable MYC-expressing populations based on sustained GFP signal (Figure 1A).

Detection and quantification of green fluorescent protein (GFP) expression, as a direct indicator of the presence of exogenous MYC, were performed using flow cytometry, allowing for a quantitative and qualitative analysis of MYC expression in MCF7-exoMYC (stable cells) (Figure 1B,C), facilitating an understanding of MYC protein levels and distribution within the studied cell population. 88.41% of MCF7 cells showed stable expression of GFP and thereby also exogenous MYC (Figure 1C), which was deemed an adequate percentage to serve as a basis for subsequent transfection assays, using the wild-type MCF7 as a reference to compare the effects of therapeutic strategies on MYC dosage compensation.

To assess whether the presence of exogenous MYC induces a change in endogenous MYC expression at the transcriptional level in the MCF7-exoMYC population, we performed RNA extraction to confirm endogenous MYC expression by RT-qPCR (Figure 1D). We compared the endogenous MYC expression of the MCF7-exoMYC with the MYC wild-type to estimate the level of MYC compensation, as the presence of the exogenous MYC leads to the downregulation of its endogenous counterpart (Figure 1D). This decrease could be explained by the compensatory pressure exerted by the exogenous MYC gene introduced into this stable cell line, as it enhances the expression of miR-17-92 cluster but lacks the 3′UTR sequence and therefore any related miRNA-mediated regulatory mechanisms. The data presented in Figure 1D indicates that endogenous MYC is downregulated in MCF7-exoMYC. This finding suggests that a stable tug-of-war system for MYC dosage compensation is feasible, providing a foundation for further investigations into strategies to disrupt this mechanism in cancer.

### 3.2. In Silico Design of miRNA-Sponge Systems Against the Members of miR-17-92 Cluster Potentially Involved in MYC Dosage Compensation

**Figure 2:**
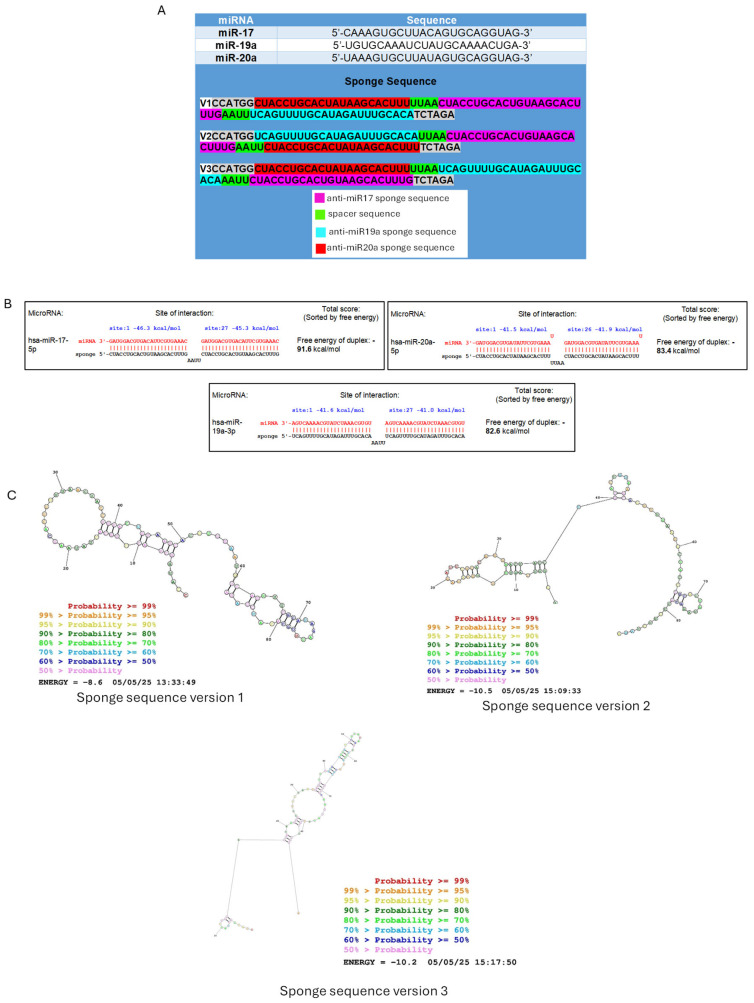
Designed sponge sequences, physicochemical properties, and evaluation of their specificity for miR-17, miR-19a, and miR-20a. (**A**) Sequences of the three versions of the sponge sequence. (**B**) Strong binding interaction between miRNAs and their corresponding sponge sequences, with binding energies represented in kcal/mol, indicating the thermodynamic stability of the miRNA-sponge complexes. (**C**) Prediction analysis of the secondary structure of the anti-miR-17, anti-miR-19a, and anti-miR-20a sponge sequences using the “RNAstructure” Server.

Using the miRNAsong software (MicroRNA SpONge Generator and tester https://www.med.muni.cz/histology/miRNAsong/ accessed on 18 February 2025), the sponge sequence was designed following the approach taking into consideration one antisense binding site for miR-17 (5′-CAAAGUGCUUACAGUGCAGGUAG-3′), miR-19a (5′-UGUGCAAAUCUAUGCAAAACUGA-3′), and miR-20a (5′-UAAAGUGCUUAUAGUGCAGGUAG-3′). Additionally, cloning sites for NcoI and XbaI restriction enzymes were integrated at each end, allowing subsequent insertion into the expression vector’s multiple cloning site to ensure directional cloning (Figure 2A).

The designed anti-miR-17, anti-miR-19a, and anti-miR-20a sponge sequences were analyzed using the miRNAsong software from Masaryk University. All analyses were performed using the following parameters: make a bulge: none; number of miRNA binding sites in a sequence: 2; spacer sequence between sponges: AAUU; energy cut-off: -25 kcal/mol; test generated sponge for potential off-targets in: Homo sapiens; seed region features: canonical 6-mer seed (positions 2–7). The resulting sponge sequences were selected based on predicted optimal binding efficiency and minimal off-target interactions, as suggested by the tool. It was found that these sponge sequences are functional against miR-17, miR-19a, and miR-20a. Full complementary interactions were observed, with ∆G values < 0, such as −91.6 kcal/mol for miR-17, −85.9 kcal/mol for miR-20a, and −82.6 kcal/mol for miR-19a (Figure 2B), indicating that the secondary structure formation process is exergonic and occurs spontaneously. However, these sequences may also serve as targets for other miRNAs at the antisense binding sites for miR-17 and miR-20a. Partial interactions were observed with ∆G values < 0 but closer to 0 (Figure S2), suggesting that the process is endergonic and not spontaneous. Nonetheless, they predominantly target miR-17, miR-19a, and miR-20a with the highest affinity.

In addition, most of the miRNAs that interact non-specifically with the binding sites of the anti-miR-17 and anti-miR-20a sponges are the same and belong to the miR-17/92 cluster (miR-18a and miR-18b) and its two paralogs, the miR-106b/25 cluster and the miR-106a/363 cluster, respectively. This non-specific binding can be explained by the shared conserved seed sequence, which places them within the same miRNA family and, consequently, the same miRNA cluster. Notably, miR-92-1 did not appear among the potential interactions; therefore, a BLAST version 2.14.1 analysis was performed to assess the specificity of the sponge sequences for miR-17, miR-19a, and miR-20a, confirming that they do not interact with this cluster member (Figure S3).

Furthermore, we performed a secondary structure prediction analysis on the three anti-miR-17/-miR-19a/miR-20a sponge sequences using the “RNAstructure” server from the Mathews group at the University of Rochester Medical Center (URMC) [20]. The following settings were applied: temperature set to 310.15 K, maximum loop size of 30 nucleotides, maximum % energy difference (MFE, MEA) of 10, maximum number of structures (MFE, MEA) of 20, window size of 3, gamma set to 1, one iteration for pseudoknot prediction, and minimum helix length of 3 for pseudoknot prediction. All other default values were left unchanged. Using these parameters allowed us to generate consistent and reliable predictions of the sponge secondary structures, ensuring proper folding and potential miRNA binding efficiency. The secondary structure analysis of the three sponge sequence versions showed the formation of stem-loop structures. The ΔG values obtained from the sequence prediction analysis were negative but closer to 0 (−8.6, −10.2, and −10.5) for versions 1, 2, and 3, respectively, indicating that this process is thermodynamically unfavorable, and there is a low probability of these secondary structures existing (Figure 2C).

Additionally, a comparison was made with the secondary structure obtained using the “RNAfold” web server from the University of Vienna [21]. This analysis confirmed the potential formation of some stem-loop structures; however, the ΔG values were −10.64, −11.38, and −11.71 (Figure S4A) for sponge sequence versions 1, 2, and 3, respectively, corroborating the results from the previous analysis.

Since miR-17, miR-19a and miR20a were reported to redundantly mediate MYC dosage compensation [8], our results suggest that a potential sponge system against those three miRNAs may robustly target miRNA-mediated MYC dosage compensation with a high sensitivity.

### 3.3. miRNA-Sponges Against the Members of miR-17-92 Cluster Revert MYC Dosage Compensation in a Stable Genetic Tug-of-War System

In order to evaluate the quality of sequences of the sponge systems, sequencing services were requested from GeneScript USA, using the recombinant plasmid pSBbi-Sponge Sequence-mCardinal-P2A-T2A-Puro in its three versions. The nucleotide sequence was analyzed via local DNA alignment using the online EMBOSS Water tool (https://www.ebi.ac.uk/Tools/psa/emboss_water/ accessed on 18 February 2025) and was compared to the in silico nucleotide sequence of the constructed pSBbi-Sponge Sequence-mCardinal-P2A-T2A-Puro plasmid. The alignment results showed 100% identity for each of the three plasmid versions, respectively (Figure S4B). These results confirm the presence of the sequences of interest in the recombinant plasmid pSBbi-Sponge Sequence-mCardinal-P2A-T2A-Puro and exclude possible mutations that could impact the expression of the anti-miR-17, anti-miR-19a, and anti-miR-20a sponge sequences in subsequent assays.

**Figure 3:**
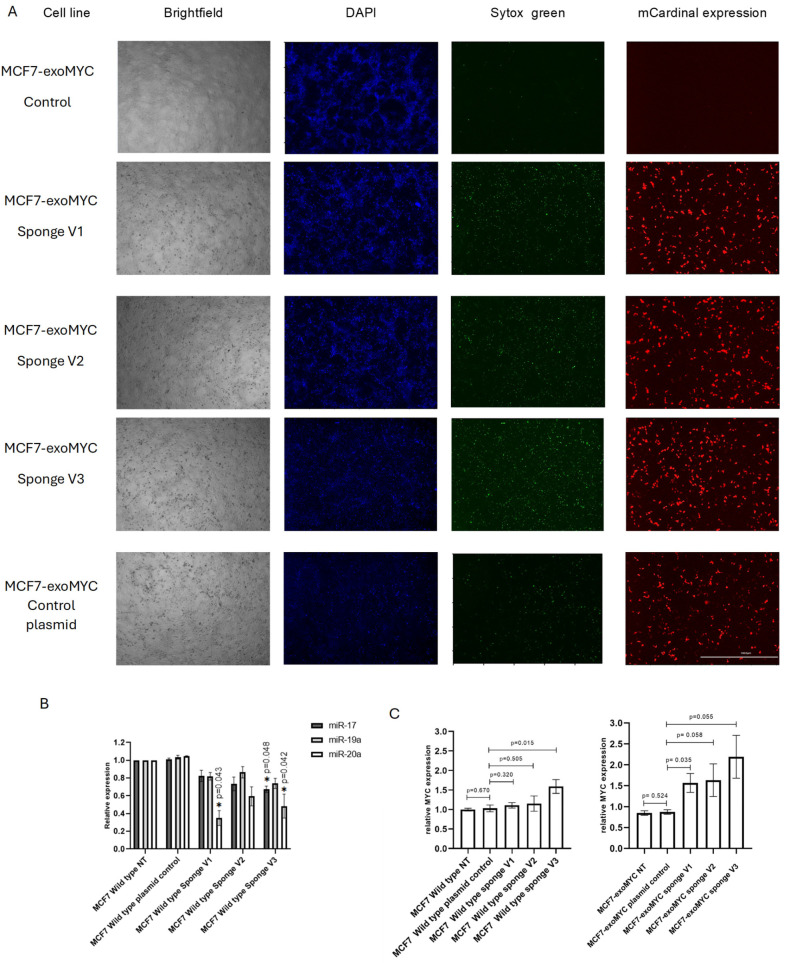
miRNAs sponges revert tug-of-war effect in MCF7 breast cancer cells. (**A**) Expression of the mCardinal reporter gene at 36 h post-transfection in MCF7-exoMYC, transfected with the recombinant plasmid pSBbi-anti-miR17, anti-miR19a, and anti-miR20a-mCardinal-P2A-T2A-Puro, and with the control plasmid pSBbi--mCardinal-P2A-T2A-Puro. Scale bar: 1000 µm. (**B**) Quantitative RT-PCR detection of hsa-miR-17, hsa-miR-19a, and hsa-miR-20a in MCF7 WT- transfected cells (*n* = 3), *t* tests were performed to analyze means of samples with a significance defined by an α of 0.05. Data is represented as mean ± SEM. The resulting *p* values were corrected for multiple comparisons using the Holm-Sidak method. (**C**) Comparison of the relative expression levels of endogenous MYC in each cell line treated with the three different sponge sequence variants and the control plasmid, normalized to the expression of the control plasmid. MCF7-exoMYC exhibiting the tug-of-war effect shows a significantly higher increase in endogenous MYC expression compared to WT cells.

Following this validation, the expression of the antimiR-17, antimiR-19a, and antimiR-20a sponge was indirectly evaluated by measuring the expression of mCardinal, the reporter gene included in the pSBbi-Sponge Sequence-mCardinal-P2A-T2A-Puro plasmid. For transfection, the recombinant plasmid or the control plasmid were introduced into MCF7 wild type cells and MCF7-exoMYC. Transfection efficiency was monitored in real time using the Cytation 5 imaging system, capturing images every 12 h. The highest transfection efficiency was observed at 36 h post-transfection in both wild-type MCF7 cells (Figure S5) and MCF7-exoMYC cells (Figure 3A).

To confirm the targeting of our sponge system, we evaluated the expression of the three miRNAs predicted by the minimal MYC dosage compensation model previously described in our study [8]. Total RNA was extracted from cells treated with the three sponge variants or the control plasmid 36 h post-transfection, and miRNA expression was quantified by RT-qPCR (Figure 3B). Our results showed significant reductions in miRNA levels, suggesting that the sponge sequences successfully bound to these miRNAs. Additionally, significant differences were observed in miR-17 expression with sponge version 1, as well as in miR-17 and miR-20a expression with sponge version 3 (Figure 3B). These findings support and align with the bioinformatics analysis of sponge specificity (Figure 2B), as the designed sponges for miR-17 and miR-20a can sequester not only their specific miRNA but also other closely related targets. Specifically, the miR-17 sponge can capture both miR-17 and miR-20a, while the miR-20a sponge can sequester both miR-20a and miR-17.

To determine whether our systems can interfere with MYC dosage compensation at the transcriptional level, we extracted RNA from cells transfected with either the recombinant pSBbi-Sponge Sequence-mCardinal-P2A-T2A-Puro plasmid or the control plasmid. A notable increase in endogenous MYC expression was observed in MCF7-exoMYC cell lines treated with the three sponge variants compared to wild type cell lines. This finding supports our initial hypothesis, indicating that the suppression of miRNAs mediating MYC dosage compensation leads to its upregulation, particularly in cell lines subjected to exogenous MYC expression pressure (Figure 3C). It also indicates that our stable tug-of-war system is sensitive to perturbation of those circuits, confirming thereby its potential application to screen or test for therapeutic strategies aiming to interfere with gene dosage compensation.

### 3.4. A miRNA Sponge System Against the Members of miR-17-92 Cluster Leads to MYC Dosage-Sensitive Cytotoxicity

**Figure 4:**
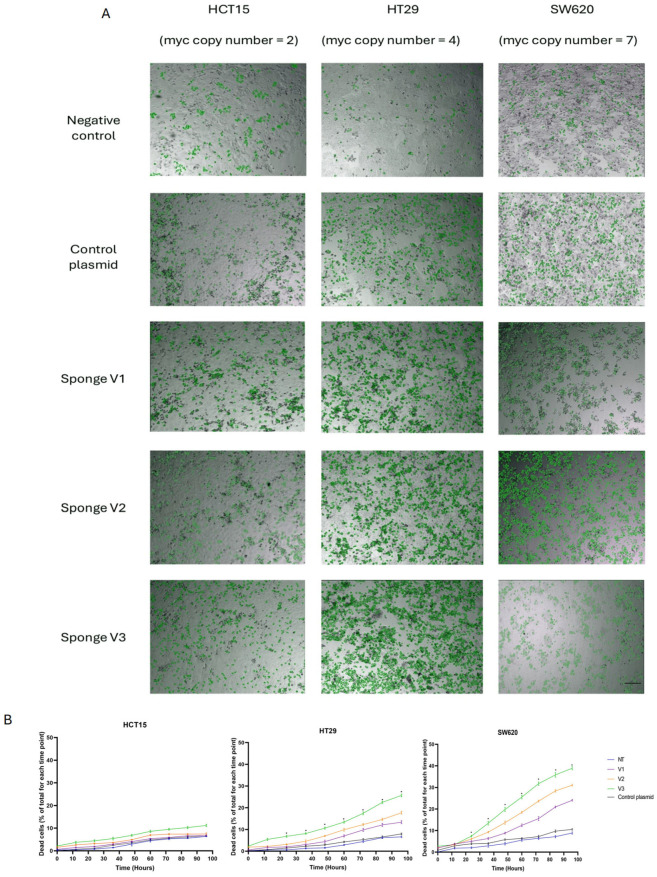
A miRNA sponge system against miR-17-92 members reveals cytotoxicity linked to MYC levels. (**A**) Inhibition of miR-17, miR-19a, and miR-20a in colon cancer cell lines with low, medium, and high MYC copy numbers reveals a differential sensitivity to disruption of MYC gene dosage compensation. Specifically, cell lines with MYC amplification exhibit increased cytotoxicity upon miRNA inhibition, suggesting a dependency on dosage compensation mechanisms for survival. Representative brightfield images highlight dead cells, indicated by green-circled regions Quantitative analysis was performed using an automated image analysis pipeline in CellProfiler. Scale bar: 100 µm. (**B**) Quantification of cell death shows a significant increase in the number of dead cells in HT29 and SW620 cell lines treated with the three sponge variants, compared to cells transfected with the control plasmid (*n* = 3; data presented as mean ± SEM). Statistically significant differences were detected after 12 h of incubation, particularly, V2 and V3 exhibited highly significant differences as early as 12 h (*p* = 0.000001 and *p* = 0.003547, respectively), suggesting a rapid onset of cytotoxic effects. This trend continued over time, with *p*-values remaining below 0.01 for nearly all time points beyond 24 h across all sponge variants).

We next examined whether the miRNAs sponge system reported above to block MYC dosage compensation is also able to induce cell death and if the amount of MYC copies influences the cellular susceptibility to this blockade. For this purpose, three colon cancer cell lines from the NCI-60 panel with different MYC gene copy numbers were used. Conditions used to transfect sponges and plasmid control in the MCF7 cancer cell line were applied to the three colon cancer cell lines: HCT-15 (2 copies of MYC), HT-29 (4 copies of MYC), and SW-620 (7 copies of MYC). The cells were incubated and monitored by live microscopy for 96 h in the presence of Hoechst to stain all nuclei, and sytox green to stain dead cells. The images were analyzed using CellProfiler [22] to quantify the number of live and dead cells over time (Figure 4A). Qualitative observation of the images did not show differences in growth or cell death in the HCT-15 cells transfected with anti-miR and control cells. However, a slight difference was observed between these two conditions in HT-29 cells, and a much more pronounced difference in SW-620 cells (Figure 4A).

Quantification of cell death in three replicates revealed a significant increase in SW-620 cells treated with version 3 of the sponge compared to the control plasmid (Figure 4B). These results indicate that cells containing a higher number of MYC copies are more vulnerable to the blockade of gene dosage compensation due to the disruption of the circuits mediated by miR-17, miR-19a, and miR-20a. Moreover, we hypothesize that aneuploid cancer cells with a higher number of copies are more susceptible to this blockade of MYC dosage compensation.

## 4. Discussion

The regulation of gene expression through microRNAs has an essential within the cellular machinery, influencing a wide range of biological processes. Identifying new interactions between miRNAs and their targets is crucial for understanding the complexity of these regulatory networks [23]. In the present study, we focused on the experimental evaluation of miRNA sponges as robust regulators of gene expression dynamics. This approach was grounded on our previous bioinformatic and systems biology analyses, where we demonstrated the role of the miR-17/92 cluster in mediating MYC dosage compensation using the NCI-60 panel, 1000 cell lines from the Cancer Cell Line Encyclopedia, and breast cancer tissue data from TCGA [8]. These prior integrative studies provided the conceptual and biological framework that guided the experimental strategies developed here.

The miR-17-92 cluster is a well-characterized oncogenic group of miRNAs that is frequently overexpressed in a wide range of cancers. It promotes tumor progression by regulating critical processes such as proliferation, apoptosis, and cellular differentiation, and is closely linked to MYC, a major oncogene. MYC directly activates the transcription of miR-17-92, which in turn sustains MYC signaling by repressing tumor suppressors and even MYC itself, forming a feedback loop that supports cancer cell survival and growth [24]. Importantly, accumulating evidence indicates that the miR-17-92 cluster also plays a key role in the maintenance and self-renewal of cancer stem cells, contributing to the stem cell origin of tumors and promoting tumor initiation and aggressiveness [11,12,13]. This dual function—driving both bulk tumor cell proliferation and sustaining stem-like properties—underscores the critical relevance of targeting this cluster for therapeutic interventions aimed at both tumor growth and cancer stem cell maintenance. This interplay, especially involving miR-17, miR-19a, and miR-20a, contributes to MYC dosage compensation in aneuploid cancer cells, allowing them to maintain oncogenic activity despite genomic instability [8]. Disrupting these regulatory loops could weaken cancer cell adaptability and represent a therapeutic opportunity.

To target the miR-17-92 cluster, anti-miRNA strategies such as anti-miRNA oligonucleotides (AMOs) and miRNA sponges have been developed. While AMOs use short complementary sequences to block miRNA activity, sponges rely on plasmid-based expression of anti-miRNA transcripts [25]. Interestingly, this approach mimics the natural function of certain circular RNAs (circRNAs) that act as endogenous miRNA sponges [26,27].

Therefore, in this study, we designed the pSBbi-Sponge Sequence-mCardinal-P2A-T2A-Puro plasmid to express a miRNA sponge sequence incorporating binding sites for miR-17, miR-19a, and miR-20a. This innovative strategy was developed as a novel approach for the specific inhibition of these miRNAs’ function with a focus on blocking MYC dosage compensation as MYC amplifications are a hallmark feature of many cancers. This could make this approach highly specific for MYC-amplified cancers while preventing toxic effects on other cells. The implementation of this strategy could offer therapeutic potential; therefore, we conducted the design of sponge systems against MYC dosage compensation along with additional experiments to validate its functionality and efficacy. Notably, the sponge sequence design was based on the methodology proposed by Kluiver [28], which is widely used for constructing sponge sequences aimed at inhibiting specific miRNAs.

The binding specificity of the sponge sequence was evaluated using miRNAsong, confirming that the anti-miR-17, anti-miR-19a, and anti-miR-20a sponge is functionally capable of inhibiting members of the miR-17-92 cluster—specifically miR-17, miR-19a, and miR-20a—through fully complementary base pairing. Additionally, the analysis demonstrated that the miR-17 sponge sequence can also bind to miR-20a and miR-20b, and vice versa, reflecting cross-binding within the same cluster, as these miRNAs share seed sequences. This feature is particularly relevant, as it enables simultaneous and coordinated inhibition of multiple related miRNAs, which could potentially enhance therapeutic efficacy by more comprehensively regulating target gene networks and amplifying the effects of intervention. Although the results also indicated that the anti-miR-17, anti-miR-19a, and anti-miR-20a sponge may bind other miRNAs at the three antisense sites, the calculated free energy (ΔG) values suggest that these interactions are weak, indicating a low probability of non-specific binding. Therefore, it is more likely that the sponge interacts with higher thermodynamic affinity with miR-17, miR-19a, and miR-20a. Notably, miRNAs that bind non-specifically to the sponge are typically overexpressed only under pathological conditions, such as cancer, minimizing the risk of off-target effects.

Thus, the establishment of the MCF7 cell line with 88.41% stable expression of the exogenous MYC sequence is a strong indicator of the effectiveness and stability of the transfection process, offering a robust and reliable platform for investigating the molecular mechanisms and cellular interactions associated with the MYC gene in the context of breast cancer. In the context of MCF7, a widely used and stable breast cancer cell line, this system is also highly useful for future research.

The relative gene expression analysis through RT-PCR revealed a significantly lower relative expression of the endogenous MYC gene in MCF7 cells with stable expression. This phenomenon could be attributed to various gene dosage compensation mechanisms activated in aneuploid cells. This is because the exogenous MYC sequence expressed by the stable cell line cannot be regulated by miRNAs, since the 3′ UTR region is absent, thereby leading to the downregulation of the endogenous version only. The identification of reduced MYC gene expression suggests the likely involvement of complex regulatory processes in response to variations in gene copy number, as previously highlighted by several authors [29,30]. However, considering the anti-miRNA sponge as a transcriptional-level regulator, it raises the possibility of fundamental compensation mechanisms at this level. These mechanisms, involving both miRNAs and transcription factors, may play key roles in more intricate regulatory network [8,31].

We also evaluated whether the previously miRNA sponge system designed to block MYC dosage compensation could additionally induce cell death, and whether MYC copy number influenced cellular susceptibility to this inhibition. To this end, we employed three colon cancer cell lines from the NCI-60 panel with different MYC copy numbers (HCT-15: 2 copies, HT-29: 4 copies, and SW-620: 7 copies), applying the same transfection conditions established in MCF7. Our results showed that inhibition by miRNA sponges had no clear effect in HCT-15, which has a low MYC copy number. However, an intermediate effect was observed in HT-29, and a significant induction of cell death in SW-620, the cell line with the highest copy number. These findings suggest that the efficacy of the sponge system directly depends on the level of MYC gene amplification, underscoring the importance of the genomic context in the therapeutic response.

These results can be explained by both the structural properties of the sponges and the specific molecular context of each cell line. Our data show that among the three constructs, Sponge V3 exerts the strongest effect on MYC regulation, representing a clear reversal of the “tug-of-war” dynamic between miRNAs and their targets. In both wild-type and stable MCF7 cells, Sponge V3 induced the highest increase in MYC expression, underscoring its enhanced capacity to sequester miR-17, miR-19a, and miR-20a and to release MYC from miRNA-mediated repression. This effect is likely due to the ability of Sponge V3 to adopt a secondary structure that renders its binding sites more accessible, thereby improving its sequestration efficiency. Notably, SW-620 cells, which carry seven copies of MYC, showed a significant increase in cell death when transfected with Sponge V3 compared to the control plasmid. This finding supports the hypothesis that aneuploid cells with higher MYC copy numbers are strongly dependent on the compensatory miR-17/92 network to maintain MYC homeostasis despite genomic instability. By disrupting this network, Sponge V3 prevents proper dosage compensation, leading to an imbalance in MYC expression and rendering these cells more susceptible to loss of viability. Overall, these data reinforce the notion that both the sponge’s secondary structure and the genomic context of MYC amplification determine the differential effects observed on proliferation and cell death. Nevertheless, although the results obtained support a link between inhibition of MYC dosage compensation and cell death, additional assays are needed to confirm that this phenomenon is a direct consequence of MYC activity. In this regard, the evaluation of MYC downstream apoptosis markers (such as BIM and caspase-3), as well as non-specific markers like Annexin V/PI, TMRM, and necroptosis/autophagy markers in time-course experiments, will allow verification of whether MYC deregulation occurs prior to apoptotic execution, thereby strengthening the evidence for a causal relationship between inhibition of this circuit and the induction of cell death [32,33,34].

The results reported in this study support the hypothesis that our miRNA sponges system is able to robustly modulate the activity of the miR-17-92 cluster, confirming the existence of a complex network of interactions between specific miRNAs [8] and their targets. This highlights the capacity of miRNA sponges to act as negative regulators of miRNA activity, suggesting a significant impact on cellular homeostasis and responses to external signals. Furthermore, our findings reveal substantial changes in gene expression associated with the interactions between miRNAs and their corresponding sponges, including a notable increase in endogenous MYC expression in both MCF7 WT and MCF7-exoMYC lines upon treatment with sponge sequences targeting miR-17, miR-19a, and miR-20a. This effect likely reflects the modulation of miRNA availability to interact with specific targets, emphasizing the role of miRNA sponges as critical regulators of key cellular pathways and gene regulatory networks.

However, it is important to acknowledge that the present study is limited to in vitro experiments. While these results provide valuable mechanistic insights, additional validation in animal models is necessary to confirm the physiological relevance and therapeutic potential of miRNA sponges in vivo. This limitation should be considered when interpreting the data, as the cellular context and systemic interactions in living organisms may influence the efficacy and specificity of miRNA sponge-mediated regulation.

In addition, certain challenges related to the delivery strategy should be considered. For instance, the current liposome-based transfection approach may exhibit insufficient efficiency in some cellular contexts, representing a bottleneck for optimal miRNA modulation. Future studies could explore alternative delivery methods, such as viral vectors or nanoparticle-based systems, to enhance uptake and efficacy in vitro and in vivo. Compared with LNA gapmeRs, miRNA sponges offer notable advantages, including the simultaneous inhibition of multiple miRNAs within a cluster and the avoidance of potential toxicity associated with complete silencing of the entire miRNA cluster. These features underscore the therapeutic potential of miRNA sponges while highlighting areas where further optimization of delivery strategies is warranted.

## 5. Conclusions

In conclusion, our findings highlight the significant role of miRNA sponges as regulators of gene expression by effectively sequestering key members of the oncogenic miR-17-92 cluster. The successful design and validation of the pSBbi-Sponge Sequence-mCardinal-P2A-T2A-Puro plasmid demonstrates the feasibility of using miRNA sponges to modulate the expression of oncogenes such as MYC, providing valuable insights into the regulatory mechanisms governing tumorigenesis. The observed reduction in miRNA expression and the associated changes in MYC regulation suggest that miRNA sponges could serve as a promising tool for therapeutic intervention. Additionally, the findings reinforce the notion of the complexity of gene regulatory networks, emphasizing the intricate interplay between miRNAs, transcription factors, and compensatory mechanisms in aneuploid cancer cells. Future studies should further explore the clinical potential of miRNA sponges against gene dosage compensation in cancer therapy, assessing their efficacy in in vivo models and their potential applications in personalized medicine

## Data Availability

The authors declare that the data supporting the findings of this study are available within the paper and its Supplementary Information files. Raw image data files of microscopy experiments are available from the corresponding author upon reasonable request.

## Supplementary Materials

The following supporting information can be downloaded at: https://www.mdpi.com/article/10.3390/cells14171384/s1, Figure S1: Schematic representation of the constructed plasmids; Figure S2: Molecular Interactions Between miRNAs and the Sponge Sequence Based on Sequence Complementarity; Figure S3. BLAST analysis of sponge sequence specificity against miR-92-1; Figure S4: Secondary structures of the three plasmid constructs and local alignments of the corresponding sponge sequence versions with the sequenced nucleotide data and Figure S5: Expression of the mCardinal reporter gene at 36 h post-transfection in wild type cell lines.

## Abbreviations

MYC	MYC proto-oncogene, basic helix-loop-helix (bHLH) transcription factor
miRNAs	microRNAs
MCF-7	Michigan Cancer Foundation-7 human breast adenocarcinoma cell line
HCT15	Human colorectal adenocarcinoma cell line HCT-15
HT-29	Human colorectal adenocarcinoma cell line HT-29
SW620	Human colorectal adenocarcinoma metastatic cell line SW620
LNA gapmeRs	Locked Nucleic Acid gapmers
BIM	BCL2-like 11 (apoptosis facilitator)
mTORC1	mammalianTarget of Rapamycin Complex 1
LKB1	Serine/Threonine Kinase 11
AMPK	activated protein kinase
TSC1	Tuberous Sclerosis Complex 1
mRNA	Messenger RNA
RPMI	Roswell Park Memorial Institute
FBS	Fetal Bovine Serum
exo-MYC	Exogenous version of MYC
MCF7-exoMYC	MCF7 cells stably expressing the exogenous MYC gene
GFP	Green Fluorescent Protein
circRNAs	Circular RNAs
