# Supplementary material for: A Synthetic Sponge System Against miRNAs of the miR-17/92 Cluster Targets Transcriptional MYC Dosage Compensation in Aneuploid Cancer
Source: Cells. 2025 Sep 4;14(17):1384. doi: 10.3390/cells14171384 (PMC12427730; doi:10.3390/cells14171384)
Supplement: Supplementary file 1 [file cells-14-01384-s001.zip › cells-3831767-supplementary.pdf]

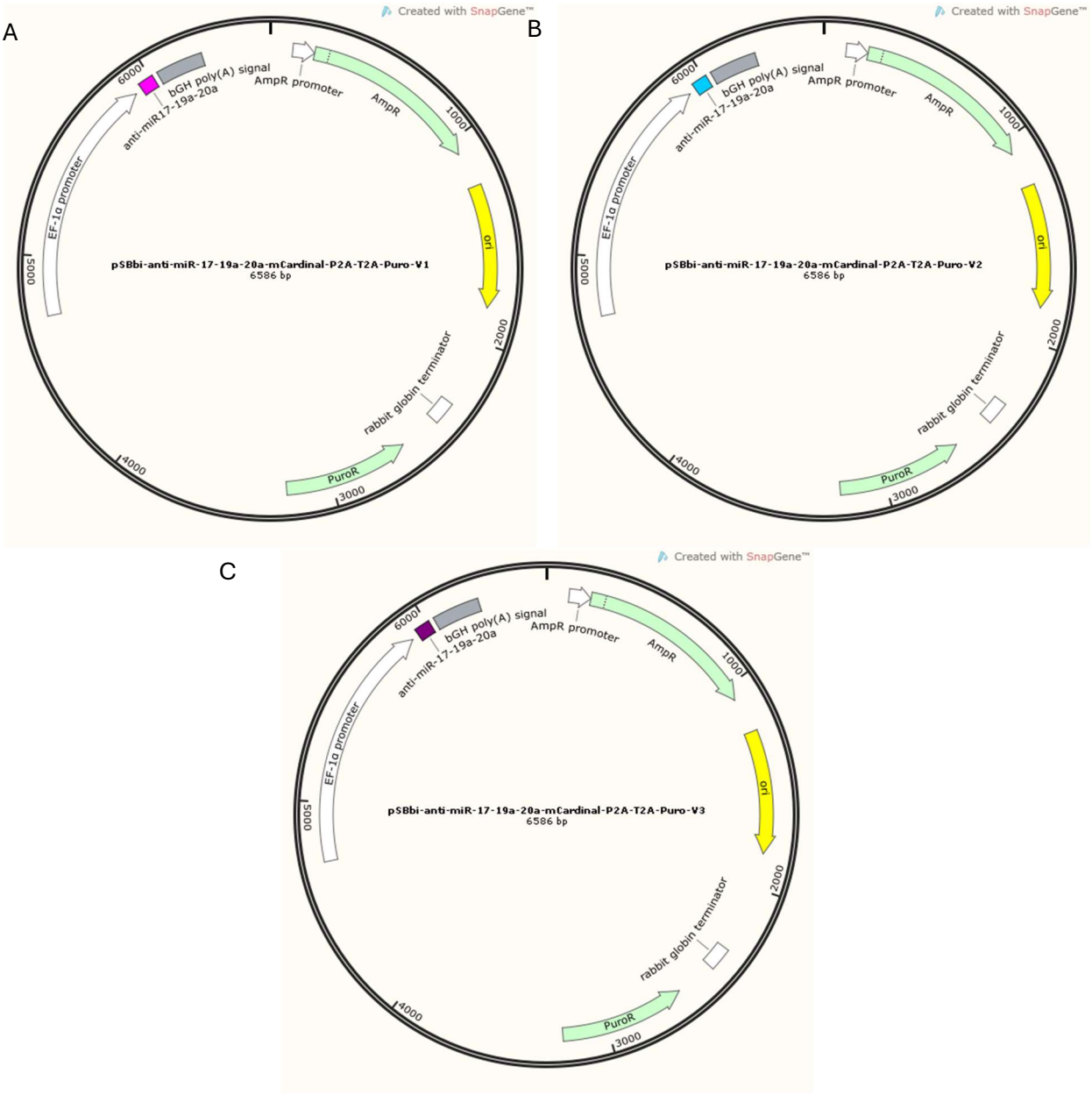

Figure S1: Schematic representation of the constructed plasmids: (A) pSBbi-anti-miR-17-19a-20a-mCardinal-P2A-T2A-Puro-V1, (B) pSBbi-anti-miR-17-19a-20a-mCardinal-P2A-T2A-Puro-V2, and (C) pSBbi-anti-miR-17-19a-20a-mCardinal-P2A-T2A-Puro-V3. Each map illustrates the organization and orientation of the inserted genetic elements, including the anti-miR sequences, and puromycin resistance marker. Maps were generated using SnapGene software.

miR-17

MicroRNA:	Site of interaction:	Total score: (Sorted by free energy)
hsa-miR-17-5p	site:1 -46.3 kcal/mol site:27 -45.3 kcal/mol<br>miRNA 3'-GAUGGACGUGACAUUCGUGAAAC GAUGGACGUGACAUUCGUGAAAC<br>sponge 5'-CUACCUGCACUGUAAGCACUUUG CUACCUGCACUGUAAGCACUUUG<br>AAUU	Free energy of duplex: -**91.6** kcal/mol
hsa-miR-106a-5p	site:1 -44.1 kcal/mol site:27 -44.2 kcal/mol<br>A A<br>miRNA 3'-GAUGGACGUGACAUUCGUGAAA GAUGGACGUGACAUUCGUGAAA<br>sponge 5'-CUACCUGCACUGUAAGCACUUU CUACCUGCACUGUAAGCACUUU<br>GAAUU G	Free energy of duplex: -**88.3** kcal/mol
hsa-miR-20a-5p	site:1 -43.0 kcal/mol site:27 -42.3 kcal/mol<br>miRNA 3'-GAUGGACGUGAUAUUCGUGAAAU GAUGGACGUGAUAUUCGUGAAAU<br>sponge 5'-CUACCUGCACUGUAAGCACUUUG CUACCUGCACUGUAAGCACUUUG<br>AAUU	Free energy of duplex: -**85.3** kcal/mol
hsa-miR-20b-5p	site:1 -40.5 kcal/mol site:27 -39.5 kcal/mol<br>C C<br>miRNA 3'-GAUGGACGUGAUA UCGUGAAAC GAUGGACGUGAUA UCGUGAAAC<br>sponge 5'-CUACCUGCACUGU AGCACUUUG CUACCUGCACUGU AGCACUUUG<br>A AAUU A	Free energy of duplex: -**80** kcal/mol
hsa-miR-93-5p	site:1 -35.8 kcal/mol site:27 -34.8 kcal/mol<br>CUUG CUUG<br>miRNA 3'-GAUGGACGUG UCGUGAAAC GAUGGACGUG UCGUGAAAC<br>sponge 5'-CUACCUGCAC AGCACUUUG CUACCUGCAC AGCACUUUG<br>UGUA AAUU UGUA	Free energy of duplex: -**70.6** kcal/mol
hsa-miR-106b-5p	site:4 -33.5 kcal/mol site:31 -32.7 kcal/mol<br>UA G UA G<br>miRNA 3'- GACGUGACA UCGUGAAAU GACGUGACA UCGUGAAAU<br>sponge 5'- CUGCACUGU AGCACUUUG CUGCACUGU AGCACUUUG<br>CUA C A AAUUCUA C A	Free energy of duplex: -**66.2** kcal/mol
hsa-miR-18a-5p	site:1 -27.7 kcal/mol site:27 -27.0 kcal/mol<br>A CUA A CUA<br>miRNA 3'-GAU GACGUGAU CGUGGAAU GAU GACGUGAU CGUGGAAU<br>sponge 5'-CUA CUGCACUG GCACUUUG CUA CUGCACUG GCACUUUG<br>C UAA AAUU C UAA	Free energy of duplex: -**54.7** kcal/mol
hsa-miR-18b-5p	site:1 -27.7 kcal/mol site:27 -27.0 kcal/mol<br>U CUA U CUA<br>miRNA 3'-GAU GACGUGAU CGUGGAAU GAU GACGUGAU CGUGGAAU<br>sponge 5'-CUA CUGCACUG GCACUUUG CUA CUGCACUG GCACUUUG<br>C UAA AAUU C UAA	Free energy of duplex: -**54.7** kcal/mol
hsa-miR-6855-5p	site:6 -28.7 kcal/mol<br>U GA GG U<br>miRNA 3'- CGU ACA CGUGG UUUGGGGU<br>sponge 5'- GCA UGU GCACU GAAUUCUA<br>CUACCU C AA UU CCUGCACUGUAAGCACUUUG	Free energy of duplex: -**28.7** kcal/mol
hsa-miR-1229-5p	site:15 -27.8 kcal/mol<br>G AGAG GG<br>miRNA 3'- CG GG UUUGGGAUGGGUG<br>sponge 5'- GC CU GAAUUCUACCUGC<br>CUACCUGCACUGUAA A UU ACUGUAAGCACUUUG	Free energy of duplex: -**27.8** kcal/mol
hsa-miR-197-5p	site:3 -27.0 kcal/mol<br>A G G<br>miRNA 3'- GG G GUGACG GAG AGAUGGGC<br>sponge 5'- CC U CACUGU CUU UCUACCUG<br>CUA G AAGCA UGAAU CACUGUAAGCACUUUG	Free energy of duplex: -**27** kcal/mol
hsa-miR-6511b-5p	site:10 -26.0 kcal/mol<br>G UG C C<br>miRNA 3'- ACA UCG GGG AAGA GGACGU<br>sponge 5'- UGU AGC CUU UUCU CCUGCA<br>CUACCUGCAC A A UGAA A CUGUAAGCACUUUG	Free energy of duplex: -**26** kcal/mol
hsa-miR-675-5p	site:7 -25.9 kcal/mol<br>CC G U<br>miRNA 3'- GUGACA CG GAGA GGCGUGG<br>sponge 5'- CACUGU GC UUCU CUGCACU<br>CUACCUG AA ACUUUGAA AC GUAAGCACUUUG	Free energy of duplex: -**25.9** kcal/mol
hsa-miR-1225-5p	site:3 -25.5 kcal/mol<br>G G C GC<br>miRNA 3'- GGG GUGAC CG AUGGGUG<br>sponge 5'- CCU CACUG GC UACCUGC<br>CUA G UAA ACUUUGAAUUC ACUGUAAGCACUUUG	Free energy of duplex: -**25.5** kcal/mol
hsa-miR-3621	site:3 -25.5 kcal/mol<br>GG C<br>miRNA 3'- GGACG UC UGG C UGGGCG<br>sponge 5'- CCUGC AG ACU G ACCUGC<br>CUA ACUGUA C UU AAUUCU ACUGUAAGCACUUUG	Free energy of duplex: -**25.5** kcal/mol
hsa-miR-6831-5p	site:2 -25.4 kcal/mol<br>C G A A<br>miRNA 3'- UGGA G GG GUG UGAGAUGGAU<br>sponge 5'- ACCU C CU CAC AUUCUACCUG<br>CU G A GUAAG UUUGA CACUGUAAGCACUUUG	Free energy of duplex: -**25.4** kcal/mol

miR-20a

MicroRNA:	Site of interaction:	Total score: (Sorted by free energy)
hsa-miR-20a-5p	site:1 -41.5 kcal/mol site:26 -41.9 kcal/mol<br>U U<br>miRNA 3'-GAUGGACGUGAUAUUCGUGAAA GAUGGACGUGAUAUUCGUGAAA<br>sponge 5'-CUACCUGCACUAUAAGCACUUU CUACCUGCACUAUAAGCACUUU<br>UUAA	Free energy of duplex: -**83.4** kcal/mol
hsa-miR-106a-5p	site:1 -38.1 kcal/mol site:26 -37.9 kcal/mol<br>C C A<br>miRNA 3'-GAUGGACGUGA AUUCGUGAAAA GAUGGACGUGA AUUCGUGAAA<br>sponge 5'-CUACCUGCACU UAAGCACUUUU CUACCUGCACU UAAGCACUUU<br>A UAA A	Free energy of duplex: -**76** kcal/mol
hsa-miR-20b-5p	site:1 -37.7 kcal/mol site:26 -38.1 kcal/mol<br>C C C C<br>miRNA 3'-GAUGGACGUGAUA UCGUGAAA GAUGGACGUGAUA UCGUGAAA<br>sponge 5'-CUACCUGCACUAU AGCACUUU CUACCUGCACUAU AGCACUUU<br>A UUAA A	Free energy of duplex: -**75.8** kcal/mol
hsa-miR-17-5p	site:1 -37.5 kcal/mol site:26 -37.9 kcal/mol<br>C C C C<br>miRNA 3'-GAUGGACGUGA AUUCGUGAAA GAUGGACGUGA AUUCGUGAAA<br>sponge 5'-CUACCUGCACU UAAGCACUUU CUACCUGCACU UAAGCACUUU<br>A UUAA A	Free energy of duplex: -**75.4** kcal/mol
hsa-miR-93-5p	site:1 -33.0 kcal/mol site:26 -33.4 kcal/mol<br>CUUG C CUUG C<br>miRNA 3'-GAUGGACGUG UCGUGAAA GAUGGACGUG UCGUGAAA<br>sponge 5'-CUACCUGCAC AGCACUUU CUACCUGCAC AGCACUUU<br>UAUA UUAA UAUA	Free energy of duplex: -**66.4** kcal/mol
hsa-miR-106b-5p	site:4 -27.1 kcal/mol site:30 -27.0 kcal/mol<br>UA CAG U UA CAG U<br>miRNA 3'- GACGUGA UCGUGAAA GACGUGA UCGUGAAA<br>sponge 5'- CUGCACU AGCACUUU CUGCACU AGCACUUU<br>CUA C AUA UUAACUA C AUA	Free energy of duplex: -**54.1** kcal/mol
hsa-miR-18a-5p	site:1 -26.4 kcal/mol site:26 -26.9 kcal/mol<br>A C A U A CUA U<br>miRNA 3'-GAU GACGUGAU U CGUGGAA GAU GACGUGAU CGUGGAA<br>sponge 5'-CUA CUGCACUA A GCACUUU CUA CUGCACUA GCACUUU<br>C U A UUAA C UAA	Free energy of duplex: -**53.3** kcal/mol
hsa-miR-18b-5p	site:1 -26.4 kcal/mol site:26 -26.9 kcal/mol<br>U C A U U CUA U<br>miRNA 3'-GAU GACGUGAU U CGUGGAA GAU GACGUGAU CGUGGAA<br>sponge 5'-CUA CUGCACUA A GCACUUU CUA CUGCACUA GCACUUU<br>C U A UUAA C UAA	Free energy of duplex: -**53.3** kcal/mol

miR-19a

MicroRNA:	Site of interaction:	Total score: (Sorted by free energy)
hsa-miR-19a-3p	site:1 -41.6 kcal/mol site:27 -41.0 kcal/mol<br>C<br>miRNA 3'-AGUCAAAACGUAUCUAAACGUGU AGUCAAAACGUAUCUAAACGUGU<br>sponge 5'-UCAGUUUUGCAUAGAUUUGCACA UCAGUUUUGCAUAGAUUUGCACA<br>AAUU	Free energy of duplex: -**82.6** kcal/mol
hsa-miR-19b-3p	site:1 -37.1 kcal/mol site:27 -36.5 kcal/mol<br>C C<br>miRNA 3'-AGUCAAAACGUA CUAAACGUGU AGUCAAAACGUA CUAAACGUGU<br>sponge 5'-UCAGUUUUGCAU GAUUUGCACA UCAGUUUUGCAU GAUUUGCACA<br>A AAUU A	Free energy of duplex: -**73.6** kcal/mol

Figure S2: Molecular Interactions Between miRNAs and the Sponge Sequence Based on Sequence Complementarity. Left to right :16 potential interactions with miRNAs at the antisense site designed for miR-17 of the sponge sequence in Homo sapiens; 9 potential interactions with miRNAs at the antisense site designed for miR-20a of the sponge sequence in Homo sapiens; 2 interactions with miRNAs at the antisense site designed for miR-19a of the sponge sequence in Homo sapiens, all of them using a duplex free energy cutoff of -25 kcal/mol.

A

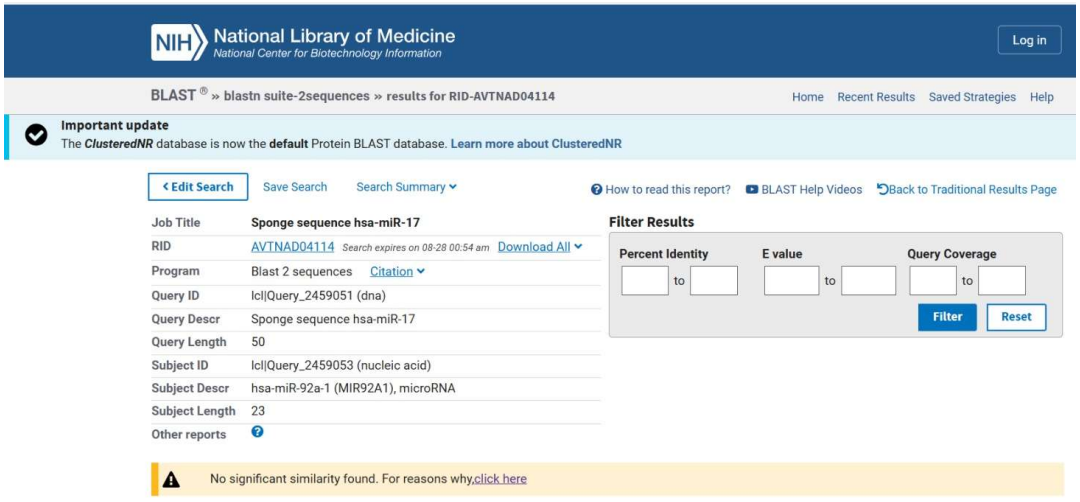

B

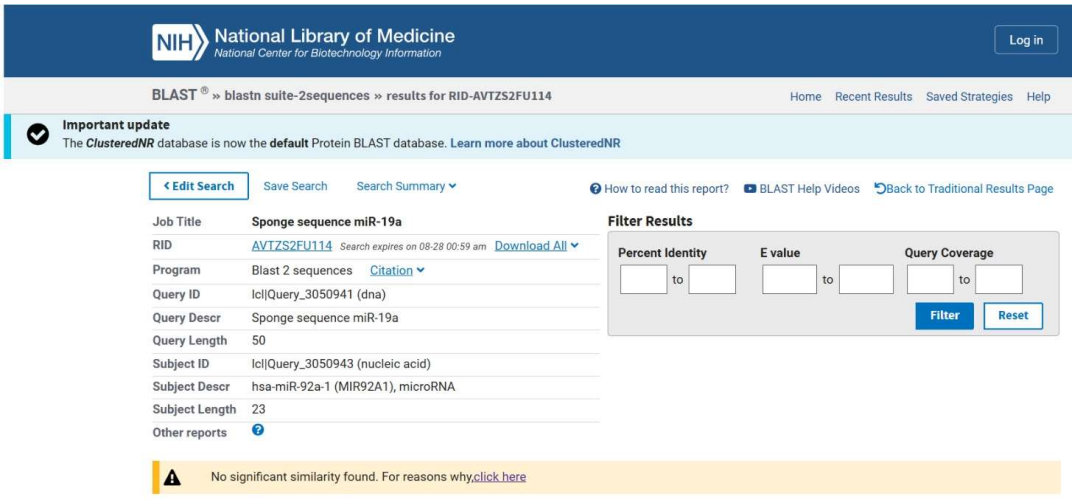

C

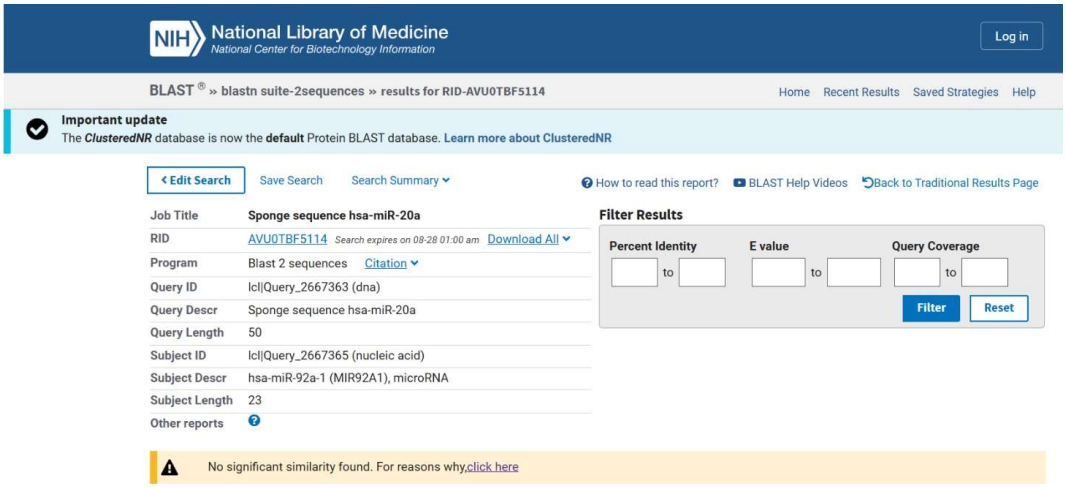

Figure S3: BLAST analysis of sponge sequence specificity against miR-92-1.

Description: A nucleotide BLAST analysis was performed to assess potential interactions between miR-92-1 and the sponge sequences designed for miR-17, miR-19a, and miR-20a. The results showed no significant alignments or complementary matches that would suggest interaction, confirming that the sponge sequences maintain high specificity for their target miRNAs within the miR-17/92 cluster. This analysis complements the partial interaction

results reported in Figure S1, ensuring that miR-92-1 is not affected by the designed constructs. Panel A shows the analysis for the miR-17 sponge sequence, panel B for the miR-19a sponge sequence, and panel C for the miR-20a sponge sequence.

A

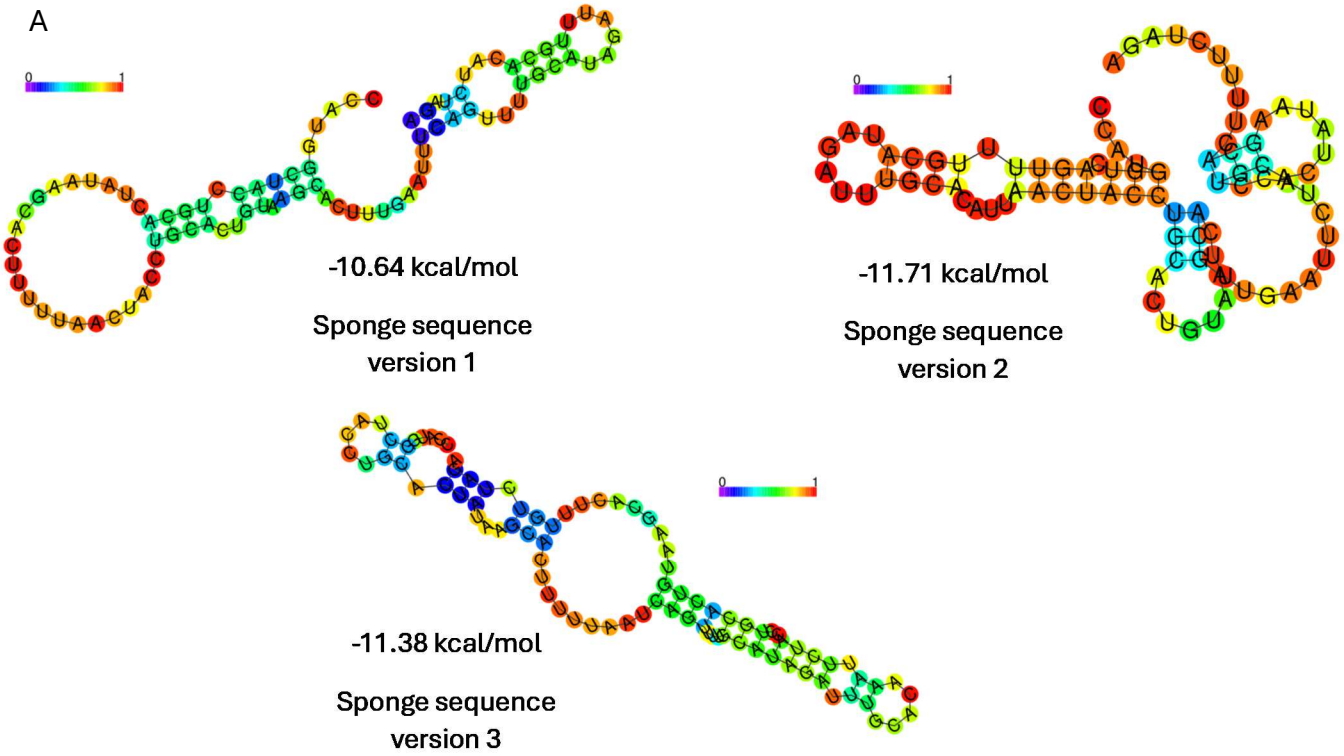

B

```
#=====================================
#
# Aligned_sequences: 2
# 1: Seq V1
# 2: pSBbi-Anti-miR-17-19a-20a-mCardinal-Puro-V1
# Matrix: EDNAFULL
# Gap_penalty: 10.0
# Extend_penalty: 0.5
#
# Length: 619
# Identity:     619/619 (100.0%)
# Similarity:   619/619 (100.0%)
# Gaps:           0/619 ( 0.0%)
# Score: 3095.0
#
#
#=====================================
```

```
#=====================================
#
# Aligned_sequences: 2
# 1: Seq V2
# 2: pSBbi-Anti-miR-17-19a-20a-mCardinal-Puro-V2
# Matrix: EDNAFULL
# Gap_penalty: 10.0
# Extend_penalty: 0.5
#
# Length: 680
# Identity:     680/680 (100.0%)
# Similarity:   680/680 (100.0%)
# Gaps:           0/680 ( 0.0%)
# Score: 3400.0
#
#
#=====================================
```

```
#=====================================
#
# Aligned_sequences: 2
# 1: Seq V3
# 2: pSBbi-Anti-miR-17-19a-20a-mCardinal-Puro-V3
# Matrix: EDNAFULL
# Extend_penalty: 0.5
#
# Length: 842
# Identity:     842/842 (100.0%)
# Similarity:   842/842 (100.0%)
# Gaps:           0/842 (0.0%)
# Score: 3487.0
#
#
#=====================================
```

Figure S4: Secondary structures of the three plasmid constructs and local alignments of the corresponding sponge sequence versions with the sequenced nucleotide data.
A) Prediction analysis of the secondary structure of the anti-miR-17, anti-miR-19a, and anti-miR-20a sponge sequences using the "RNAfold" Server.
B) Summary of the in silico local alignment of each plasmid version containing the sponge sequences with the nucleotide sequence obtained by automated sequencing.

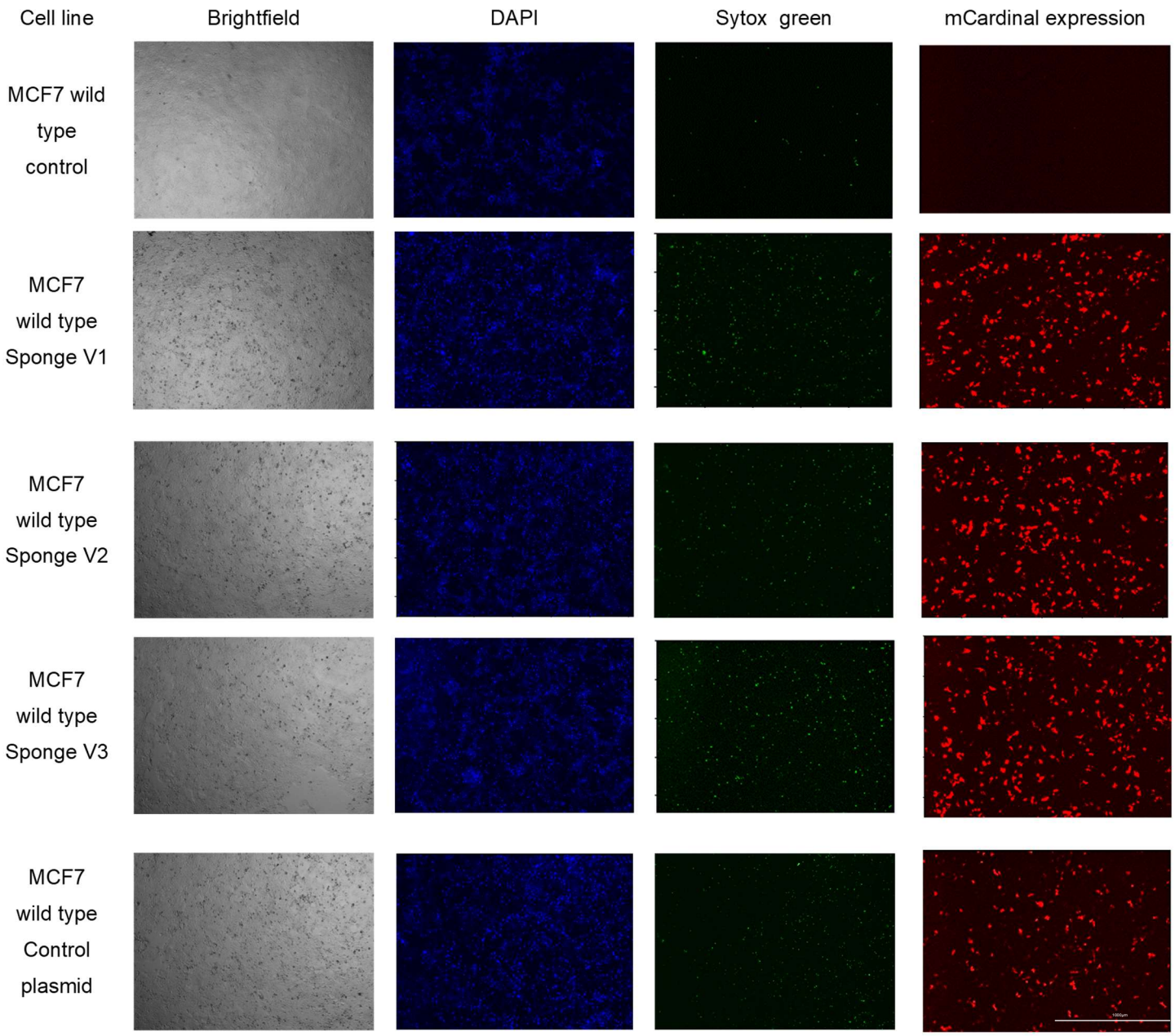

Figure S5: Expression of the mCardinal reporter gene at 36 hours post-transfection in wild type cell lines. Cells were transfected with the recombinant plasmid pSBbi-anti-miR17, anti-miR19a, and anti-miR20a-mCardinal-P2A, and with the control plasmid pSBbi-T2A-mCardinal-P2A
